# A Case of Enormous Fibro-Cystic Tumor of the Uterus

**Published:** 1889-10

**Authors:** X. O. Werder

**Affiliations:** Pittsburg, Pa.


### A CASE OF ENORMOUS FIBRO-CYSTIC TUMOR OF THE UTERUS: LAPAROTOMY. RECOVERY.

DR. X. O. WERDER, PITTSBURGH.

June 14 last, I was consulted by Miss W. C., aged 23, in regard to an abdominal tumor which had been growing for the last eight months. The abdomen was enlarged to the size of pregnancy at full term, and below the umbilicus were a number of striæ, such as you find at the end of gestation. The right side of the abdomen was more rounded than the left, the tumor apparently having developed from that side. Dullness extended from the pubes to the ensiform cartilage, and to both hypochondriac regions, leaving a tympanitic area in both flanks. The tumor was uniform, soft and elastic, and distinctly fluctuating. The largest circumference of the abdomen was between the umbilicum and pubis. Digital examination per vagina revealed a normal nulliparous cervix, high up in vagina; fundus uteri could not be felt; sound was not introduced. Vaginal examination very difficult and painful on account of a rather rigid hymen. Examination per rectum negative.

Miss C. first menstruated at 13 years, always regular and painless, lasting five days. Has been more profuse since appearance of tumor than before. Had typhoid fever five years ago, then dyspepsia for a time after, but not since. Was confined to bed with rheumatism 8 weeks; otherwise in good health. No sign of tumor until 8 months ago, and then it was only noticeable about two weeks before each menstruation, when she felt bloated; this feeling always disappeared before the next menstrual period. She was not conscious of the tumor, however, until two or three months before the operation. The tumor never gave her any trouble except about two weeks after each menstruation, when it always became distinctly larger, making her quite uncomfortable. From this history, and from the fact of the rapid growth of the tumor, and its apparent development from the right side, I did not hesitate to pronounce it an ovarian cyst. Several physicians of ability, who had examined the case before I did, had already made the same diagnosis.

June 24th I opened her abdomen at Mercy hospital. The tumor was covered by a fold of the peritoneum, traversed by a large number of blood vessels, some of them of enormous size, and studded by numerous small cysts. It was at first rather difficult to determine whether this was the omentum adherent to the tumor, or some other structure, but on pushing my hands down between the tumor and the abdominal walls, I could feel it spread out on each side of the tumor like two wings. There was, therefore, no doubt that it was the broad ligament which was enveloping the lower portion of the tumor. This fact, in addition to the tumor—which, though even now distinctly fluctuating, seemed to have a thick-set muscular wall, a fold of which, picked up between the thumb and finger, felt like the walls of an enlarged pregnant uterus—made it evident that the growth was something else than an ovarian cyst : that it was either a pregnant uterus or a soft tumor growing from the uterus. A hypodermic needle was passed into the tumor, but nothing but a few drops of serous fluid was withdrawn. Pregnancy was excluded on account of her regular menstruation, rigid hymen and virginal condition of vagnia and cervix, appearance of her breasts, absence of fœtal sounds and ballottement, etc. Concluding that we had to deal with a myoma, we proceeded to deliver it. This, however, was a difficult task. The abdominal incision was enlarged above and below, some adhesions to bowels and abdominal parietes were tied off as they were encountered, but all attempts to deliver the tumor failed until the incision had been carried up to the ensiform cartilage. By all these manipulations a great deal of time had been consumed before the tumor had been rolled out of the abdominal cavity, and the hemorrhage was alarming because of the extremely vascular nature of the tumor. The patient had become very anæmic, and at times almost collapsed, requiring constant stimulation by hypodermic injections of whisky, of which she received from thirty to forty during the operation. The broad ligaments, the right of which reached higher on the tumor than the left, and folds of peritoneum connecting the bladder and the tumor were now separated from the tumor as far as necessary to form a pedicle, and were secured by small forceps and an elastic ligature passed tightly around the tumor, just above the attachment to the fundus uteri, and the myoma cut off above the ligature. After trimming the pedicle, which was about the size of two fists, it was brought into the lower angle of the abdominal wound, and the parietal peritoneum stitched to it just below the elastic ligature, in order to shut it off from the peritoneal cavity. The broad ligaments and the peritoneal folds, secured by the artery clamps, were also brought out below the pedicle ; the forceps, six in number, were not removed, but were wrapped in iodoform gauze. In the absence of proper instruments for extra-peritoneal treatment of the pedicle —I having been prepared for an ovariotomy only, and not for a myomectamy—I compressed the upper part of the pedicle by two large Spencer Wells' forceps, one on each side, covering their handles with iodoform gauze. They were made to take the place of the clamps ordinarily used in the treatment of the pedicle by the extra-peritoneal method. The abdominal wound was then closed by silk-worm gut sutures, deep and superficial, a glass drainage tube was inserted immediately above the pedicle, the wound was covered with iodoform, and an antiseptic dressing was applied. The pedicle was seared with the actual cautery and covered with iodoform.

The patient rallied well from the shock of the operation. On the first evening temperature was 102°, pulse, 120; afterward the temperature never reached that point again, and became normal after the first week. The patient suffered very little pain, except from flatulency the first few days; the large Spencer Wells', forceps attached to the pedicle gave her most of her trouble, as they prevented her from turning on either side, keeping her constantly in the dorsal position. The drainage tube was removed about thirty hours after the operation. The small artery forceps, attached to broad ligaments and peritoneal folds, were removed on the seventh day; large Spencer Wells' forceps on the eleventh day on one side, and on the thirteenth day on the other; the elastic ligature came away with the pedicle on the nineteenth day, leaving a large, funnel-shaped opening, covered with healthy granulations. There was never any suppuration from the pedicle, it becoming dry and mummified, but on the twelfth day a small fecal fistula formed on the side of the pedicle, discharging small quantities of fecal matter at first, which ceased, however, after about ten days, nothing but some flatus coming from it now occasionally. Abdominal incision was healed by first intention when sutures were removed on the tenth day.

There is now, eight weeks after the operation, only a small, granulating surface left at the seat of the pedicle, not larger than a five-cent piece, which is rapidly cicatrizing. The patient has gained flesh since the operation, and feels perfectly well, so that she will be able to leave for her home in Ohio in a few days.

The tumor weighed twenty-six pounds, not including the pieces cut away in trimming the pedicle; they, in connection with the blood lost during the operation, would have probably increased its weight to forty pounds. The tumor was a fibro-cyst, but contained large cystic cavities. It presented rather an œdematous, spongy condition. Even when lying on the table distinct fluctuation could be obtained, and on its cut surface small drops of serous fluid was observed, like drops of sweat. No microscopical examination of the tumor was made; I had intended to harden it in alcohol and preserve it, but the weather was intensely hot and it spoiled in a few days.

The interesting points of this case are: 1. The youth of the patient; tumors of this kind are rarely found at a less age than thirty-five. 2. The rapidity of its growth; it attained this enormous size in eight months, without causing much local or constitutional disturbance. 3. Its distinct increase in size always two weeks after each menstruation; periodical enlargement of fibroid tumors is not uncommon, but this occurs generally during menstruation. The question naturally suggests itself whether swelling of the growth in this case did not correspond to the time of ovulation.

That a diagnosis of ovarian cyst was made in this case is not surprising. The fact is that it is impossible to differentiate fibrocystic tumers of the uterus, especially when of such large size as this, from ovarian tumors, as all the physical signs are almost identical. Gusserow, in his book on fibroids of the uterus (Encyclop. Obstetrics and Gynecology), says: "The diagnosis of these tumors has only been made in the most exceptional cases, and even then has been the result of accident rather than of correct appreciation of the symptoms. Fibro-cysts so closely resemble multilocular ovarian cysts, particulary in their location and in their fluctuation, that the frequency with which they have been mistaken for ovarian tumors is not astonishing.

